# People living with undiagnosed HIV infection and a low CD4 count: estimates from surveillance data, Italy, 2012 to 2014

**DOI:** 10.2807/1560-7917.ES.2018.23.15.17-00240

**Published:** 2018-04-12

**Authors:** Vincenza Regine, Maria Dorrucci, Patrizio Pezzotti, Alessia Mammone, Chantal Quinten, Anastasia Pharris, Barbara Suligoi, the regional representatives of the National HIV Surveillance System

**Affiliations:** 1 Infectious Diseases Department, Italian National Institute of Health, Rome, Italy; 2 National Institute for Infectious Diseases ‘L. Spallanzani’, Rome, Italy; 3 European Centre for Disease Prevention and Control (ECDC), Stockholm, Sweden; 4 The regional representatives are listed at the end of the article

**Keywords:** HIV infection, surveillance data, undiagnosed HIV infection, low CD4 count, modelling

## Abstract

**Background and aims:**

Late HIV diagnosis is associated with onward HIV transmission, higher morbidity, mortality and healthcare costs. In Italy, more than half of people living with HIV were diagnosed late during the last decade, with a CD4 count < 350 cells/mm^3^ at diagnosis. We aimed to determine the number and characteristics of people living with undiagnosed HIV infection and low CD4 counts in Italy.

**Methods:**

Data on newly reported HIV diagnoses from 2012 –2014 were obtained from the national HIV surveillance system. We used the European Centre for Disease Prevention and Control HIV modelling tool to calculate the undiagnosed prevalence and yearly diagnosed fraction (YDF) in people with low CD4 count.

**Results:**

The estimated annual number undiagnosed HIV infections with low CD4 count was on average 6,028 (95% confidence interval (CI): 4,954–8,043) from 2012–2014. In 2014, most of the undiagnosed people with low CD4 count were men (82.8%), a third acquired HIV through sex between men (MSM) (35.0%), and heterosexual transmission (33.4%), respectively. The prevalence of undiagnosed HIV infection was 11.3 (95% CI: 9.3–14.9) per 100,000 residents ranging from 0.7 to 20.8 between Italian regions. Nationally the prevalence rate was 280.4 (95% CI: 173.3–450.2) per 100,000 MSM, 8.3 (95% CI: 4.9–13.6) per 100,000 heterosexual men, and 3.0 (95% CI: 1.4–5.6) per 100,000 women. The YDF was highest among heterosexual women (27.1%; 95% CI: 16.9–45.2%).

**Conclusions:**

These findings highlight the importance of improving efforts to identify undiagnosed HIV infections primarily among men, both MSM and heterosexual men.

## Introduction

Late diagnosis of HIV remains a major public health concern worldwide [1-3]. In 2015, close to half (48%) of people newly diagnosed with HIV in European countries were late presenters, with CD4 counts below 350 cells/mm^3^ at diagnosis, including 28% with advanced HIV infection (CD4 < 200 cells/mm^3^) [1]. In Italy, despite HIV testing and healthcare being free of cost for the individual, more than half of the ca 4,000 people diagnosed with HIV annually are diagnosed with a CD4 count < 350 cells/mm^3^, and ca 40% are at the symptomatic stage of infection when diagnosed [4,5].

The late diagnosis of HIV infection has negative consequences, both at the individual and population levels. People presenting late respond insufficiently to antiretroviral therapy (ART) and treating them is often complex and costly. Individuals who are in an advanced stage of immunosuppression due to HIV are at high risk of clinical events and death [6,7]. At population level, those diagnosed late are a potential source of HIV transmission for a considerable period [8]. Low CD4 count and high viral load contribute significantly to the risk of sexual transmission [9].

Given the prevalence of late diagnoses, it is likely that a considerable number of people with low CD4 counts remain undiagnosed in Italy. Mammone et al. estimated that there are 12,000–18,000 undiagnosed people living with HIV in Italy [10], although no estimate of those undiagnosed with a low CD4 count was calculated. Knowing the numbers of people living with undiagnosed HIV and with a low CD4 count could be useful in predicting the prevalence of late HIV presentation and its consequences of poor prognosis and onward transmission. Being aware of the overall numbers of those who are undiagnosed and of the respective numbers in relevant subpopulations can support the monitoring of national and local HIV prevention strategies, the revision of health policies and the allocation of economic resources to prevention and control efforts [11].

The aim of this study was to estimate the number of people living with undiagnosed HIV and a low CD4 count in Italy, to analyse the characteristics of this population and to evaluate the prevalence of undiagnosed HIV infection in the general population.

## Methods

To estimate the number of people living with undiagnosed HIV and a low CD4 count, we applied the model proposed by Lodwick et al. [12]. This model is part of the European Centre for Disease Prevention and Control (ECDC) HIV modelling tool. The tool uses routine surveillance data to calculate estimates of the number of people living with HIV as well as of those not yet diagnosed. It does not depend on historical data, i.e. it can be used even with limited years of available data [13]. Of the two models included we choose the London model as it applies to the way HIV data was collected through the Italian HIV surveillance system effective as of 2012.

### Data source

We obtained data from the Italian National HIV Surveillance system (INHS) on people aged > 15 years who were diagnosed with HIV between 2012 and 2014 and reported to the INHS by June 2015 [5].

In Italy, the reporting of new HIV diagnoses is mandatory since July 2008 for all clinicians [14]. There are 173 Infectious Diseases Centers (IDC) in Italy that offer free monitoring and health management to all HIV-positive persons, including non-nationals and undocumented migrants [15]. Most people who test HIV-positive are diagnosed at IDCs directly and those who are tested in other health facilities are further referred to an IDC for confirmatory testing and diagnosis.

Data on new diagnoses are collected by regional surveillance systems and sent annually to the INHS coordinated by the Italian national institute of health in Rome. While the surveillance did not cover all regions previously, since 2012 there was 100% geographical coverage with all regions reporting data to the INHS [5].

The INHS collects the following data on an annual basis: (i) demographic data (age, sex, nationality, geographical area of diagnosis, and geographical area of residence), (ii) clinical information (clinical stage, CD4 counts, and viral load) and (iii) HIV exposure group data (people who inject drugs (PWID), heterosexual women, heterosexual men, men who have sex with men (MSM), and other/not available) [5].

Residences in Italy were grouped into three areas based on the of Italian National Bureau of Statistics (ISTAT) classification: (i) North (Piemonte, Valle d’Aosta, Liguria, Lombardia, Trentino–Alto Adige, Friuli–Venezia Giulia, Veneto and Emilia Romagna); (ii) Central (Toscana, Marche, Lazio, and Umbria); and (iii) South (Abruzzo, Molise, Campania, Puglia, Calabria, Basilicata, Sicilia, and Sardegna) [16]. For non-Italian citizens without residence in Italy, we assigned the place of diagnosis as residence.

### Description of the model

The model proposed by Lodwick et al. [12] is based on back-calculation principles. In brief, the method assumes that people living with undiagnosed HIV develop AIDS or other HIV-related symptoms of sufficient severity, or symptoms which are sufficiently specific to HIV, and will seek care and, as a result, be diagnosed with HIV. It uses information derived from people newly diagnosed at an advanced stage of HIV infection (clinical stage B or clinical stage C) with CD4 counts < 350 cells/mm^3^, stratified into eight groups based on CD4 counts at HIV diagnosis (< 20, 20–49, 50–99, 100–149, 150–199, 200–249, 250–299, 300–350). Specifically, for each CD4 count stratum the number of those undiagnosed is obtained by dividing the number of symptomatic diagnoses by the CD4-specific rate of HIV symptoms estimated in cohort studies. The total number of undiagnosed people with HIV with a CD4 count < 200 cells/mm^3^ (advanced HIV infection) was obtained summing the specific stratum estimates, from the first (< 20 cells/mm^3^) to the fifth (150–199 cells/mm^3^) stratum. Similarly, the total number of undiagnosed people with HIV with a CD4 count < 350 cells/mm^3^ (late presenters) was obtained summing the eight specific stratum estimates.

The ECDC HIV modelling tool version 1.2.1 [13] was used to calculate the estimates stratified by main demographic characteristics (age, sex, nationality, geographical area of residence) and HIV exposure groups (PWID, heterosexual women, heterosexual men, MSM, and other/not available). The tool permits the estimation of undiagnosed people living with HIV according to the different characteristics.

### Adjustment for missing values and reporting delay

The estimates obtained from the model were adjusted for reporting delays or underreporting of HIV diagnoses with HIV-related symptoms as proposed in the original publication [12]. As information on clinical stage and CD4 count were missing in around 30% of the INHS records, the missing values were adjusted under the assumption that the distribution of clinical stage and CD4 count among diagnosed cases with missing data was similar to that of diagnosed cases with available data [17]. In brief, the estimates obtained from the London method were divided by the proportion of all diagnoses with symptoms, where the CD4 count was known.

The following steps were used for the adjustment: first, the proportion of missing data was calculated relative to the clinical stage and CD4 count for each characteristic of the new diagnoses. Second, the estimates of undiagnosed people living with HIV were multiplied by the inverse of the missing proportion according to each characteristic. Last, the reporting delay to the INHS was considered, with the adjusting of the annual estimates by a reporting delay of 5%, introduced for each year of the 3 years, given that all new HIV diagnoses were notified to the surveillance system within 3 years after diagnosis [5]. In other words, it was assumed that in 2015 the INHS data were all complete for 2012 (100%), and almost complete for 2013 (95%) and 2014 (90%). Therefore, for each characteristic we adjusted the estimates multiplying them by the following:

$$\text{ Adjustment factor }=\frac{1}{\left(1\;–\;proportion\;of\;missing\;-\;annual\;reporting\;delay\right)}$$

Table 1 shows the proportion of missing data (of CD4 count and/or clinical stage) with the respective ‘adjustment factors’ applied to undiagnosed people living with HIV estimates by main characteristics. As an example, when estimating the undiagnosed number of women, missing CD4 count and clinical stage accounted for 27% in 2012, 29% in 2013, and 26% in 2014 of the cases. The adjustment factors for women were: 1/(1 - 0.27) = 1.37; 1/(1 - 0.29 - 0.05) = 1.52 and 1/(1 - 0.26 - 0.05 - 0.05) = 1.56 in the 3 years, respectively. Of note, there was a direct relationship between the proportions of missing data and the value of the adjustment factors: the higher the adjustment factor, the higher the proportion of missing data (Table 1).

**Table 1:** CD4 count and/or clinical stage missing value proportions for HIV diagnoses and adjustment factors by specific stratum estimate for undiagnosed people living with HIV by year, Italy, 2012–2014

	2012		2013		2014	
	Missing values for CD4 count or clinical stage (%)	Adjustment^a^ factor	Missing values for CD4 count or clinical stage (%)	Adjustment^a^ factor	Missing values for CD4 count or clinical stage (%)	Adjustment^a^ factor
**Total diagnoses^b^**	29	1.40	30	1.55	29	1.64
**Sex**						
Women	27	1.37	29	1.52	26	1.56
Men	29	1.41	31	1.56	30	1.66
**Age group (years)**						
15–24	38	1.61	33	1.60	28	1.61
25–34	33	1.49	34	1.63	34	1.78
35–44	26	1.36	29	1.52	28	1.61
45–54	24	1.31	29	1.52	27	1.59
≥ 55	24	1.32	26	1.44	25	1.53
**HIV exposure group**						
PWID	18	1.23	23	1.39	21	1.46
Heterosexual women	23	1.31	25	1.42	22	1.47
Heterosexual men	25	1.34	28	1.48	29	1.64
MSM	27	1.36	26	1.46	24	1.52
Other/NA	51	2.02	64%	3.25	60	3.36
**Nationality**						
Italian	27	1.37	28	1.48	27	1.58
Non-Italian	34	1.51	39	1.79	35	1.83
**Geographical area^c^**						
North^d^	22	1.27	24	1.41	23	1.48
Central^e^	62	2.60	60	2.86	59	3.24
South	3	1.03	5	1.11	3	1.14

NA: not available; MSM: men who have sex with men; PWID: people who inject drugs.

^a^ Adjustment factors were calculated as follows = 1 / (1 - proportion of missing - annual reporting delay).

^b^ Missing values for CD4 count in 2012 were 21.7% and for clinical stage 28.2%; missing values for CD4 count in 2013 were 21.8% and for clinical stage 30.1%; missing values for CD4 count in 2014 were 22.2% and for clinical stage 28.6%.

^c^ North area includes: Piemonte, Valle d’Aosta, Liguria, Lombardia, Trentino– Alto Adige, Friuli–Venezia Giulia, Veneto, Emilia Romagna; Central area includes: Toscana, Marche, Lazio, Umbria; South area includes: Abruzzo, Molise, Campania, Puglia, Calabria, Basilicata, Sicilia, Sardegna.

^d^ In the North area, the proportion of missing values were concentrated mainly in one region that does not routinely collect data on clinical stage.

^e^ In the Central area, the proportion of missing values were concentrated mainly in one region (100% in one region and less than 5% in the remaining three regions) that does not routinely collect data on the clinical stage and CD4 count.

### Characteristics of people undiagnosed and newly diagnosed with HIV and with low CD4 count in 2014

The characteristics of both people undiagnosed and newly diagnosed with HIV and a low CD4 count were described for the year 2014 to compare characteristics of those undiagnosed with low CD4 count with new HIV diagnoses with a low CD4 count.

The yearly diagnosed fraction (YDF) in people with a low CD4 count (CD4 < 350 cells/mm^3^ or CD4 < 200 cells/mm^3^) was calculated according to main characteristics for the year 2014. YDF has been recently proposed by Sasse et al. [18] to evaluate the ratio of new diagnoses among people living with HIV who can be diagnosed in a given year. In our study, YDF was calculated among people with HIV and with a low CD4 count according to the following formula:

$$\text{YDF }=\;\frac{Number\;of\;new\;diagnoses\;}{\left(Number\;of\;new\;diagnoses\;+\;Estimated\text{ number }of\;undiagnosed\;HIV\;\right)}\text{x}100$$

### Prevalence of undiagnosed HIV infection with low CD4 count in 2014

To evaluate the prevalence of undiagnosed people living with HIV and a low CD4 count for the year 2014, the rate expressed was calculated as follows:

$\frac{Estimated\text{ number }of\;undiagnosed\text{ people living with }HIV\;with\;a\;CD4<350\;cells/mm^{3}}{Number\;of\;people\;aged\;15-74\;years}\text{ x }100,000$

As a denominator, the population aged > 15 years up to 75 years estimated by the ISTAT was used [16].The described undiagnosed prevalence of HIV infection was also calculated by region of residence and by HIV exposure group. As denominator, the female population for heterosexual women and the male population for men was used. For MSM, a proportion of 3% of the adult male population was assumed, given that published data reveals estimates of MSM ranging from 2% to 4% of the male population in Italy [19-21]. Thus, for heterosexual men the remaining 97% of male population was used.

## Results

### National HIV surveillance system data

About 4,000 new HIV diagnoses were notified to the INHS annually during the period 2012–2014 (Table 2). Clinical stage at HIV diagnosis was reported for 70% of people, 39% of them were diagnosed at clinical advanced stage (clinical stage B or C). Table 2, shows the distribution of new HIV diagnoses by main characteristics; these were similar during the 3 years: the majority were men, more than half aged between 25 and 44 years, and more than one third were MSM. More than half were diagnosed late, namely with CD4 count < 350 cells/mm^3^.

**Table 2:** Main characteristics of new HIV diagnoses in people aged above 15 years by year, Italy, 2012–2014

	2012			2013			2014		
	n	%	% excluding NA values	n	%	% excluding NA values	n	%	% excluding NA values
**Total diagnoses**	4,127	–	–	3,797	–	–	3,679	–	–
**Sex**									
Women	872	21.1	21.1	833	21.9	21.9	746	20.3	20.3
Men	3,255	78.9	78.9	2,964	78.1	78.1	2,933	79.7	79.7
**Age group (years)**									
15–24	331	8.0	8.0	291	7.7	7.7	322	8.8	8.8
25–34	1,319	32.0	32.0	1,121	29.5	29.5	1,063	28.9	28.9
35–44	1,239	30.0	30.0	1,210	31.9	31.9	1,131	30.7	30.7
45–54	827	20.0	20.0	775	20.4	20.4	746	20.3	20.3
≥ 55	411	10.0	10.0	400	10.5	10.5	417	11.3	11.3
**HIV exposure groups**									
PWID	211	5.1	5.1	178	4.7	4.7	141	3.8	3.8
Heterosexual women	704	17.1	17.1	709	18.7	18.7	625	17.0	17.0
Heterosexual men	1,059	25.7	25.7	981	25.8	25.8	973	26.5	26.5
MSM	1,579	38.2	38.2	1,507	39.7	39.7	1,512	41.1	41.1
Other/NA	574	13.9	13.9	422	11.1	11.1	428	11.6	11.6
**Nationality**									
Italian	3,019	73.1	73.6	2,864	75.4	75.6	2,671	72.6	72.9
Non-Italian	1,084	26.3	26.4	925	24.4	24.4	995	27.0	27.1
NA	24	0.6	–	8	0.2	–	13	0.4	–
**Geographical area**									
North	2,354	57.0	57.0	2,113	55.6	55.6	1,978	53.8	53.8
Central	1,054	25.6	25.6	1,028	27.1	27.1	1,023	27.8	27.8
South	719	17.4	17.4	656	17.3	17.3	678	18.4	18.4
**Clinical stage of HIV infection**									
A	1,832	44.4	61.8	1,660	43.7	62.6	1,705	46.3	64.9
B	483	11.7	16.3	397	10.5	14.9	352	9.6	13.4
C	649	15.7	21.9	596	15.7	22.5	571	15.5	21.7
NA	1,163	28.2	–	1,144	30.1	–	1,051	28.6	–
**CD4 count (cells/mm^3^)**										
< 200	1,188	28.8	36.7	1,108	29.2	37.3	998	27.1	34.9
200–349	591	14.3	18.3	587	15.5	19.8	531	14.4	18.6
350–499	581	14.1	18.0	514	13.5	17.3	535	14.5	18.7
≥ 500	873	21.2	27.0	759	20.0	25.6	798	21.7	27.9
NA	894	21.7	–	829	21.8	–	817	22.2	–

MSM: men who have sex with men; NA: not available; PWID: people who inject drugs.

### Estimates of people living with undiagnosed HIV and a low CD4 count

Using the described model, the estimated number of people living with undiagnosed HIV infection and with CD4 count < 350 cells/mm^3^ in Italy was 6,028 (95% CI: 5,090–7,826) in 2012, 6,156 (95% CI: 4,891–8,517) in 2013, and 5,899 (95% CI: 4,882–7,786) in 2014. Table 3, shows the estimated number of undiagnosed people living with HIV and CD4 count < 350 cells/mm^3^ by demographic information, and by geographical area of residence. The highest estimated numbers in 2014 were in men (4,893; 95% CI: 3,992–6,568), both MSM (2,115; 95% CI: 1,292–3,395) and heterosexual men (2,017; 95% CI: 1,183–3,301) as well as in people living in the North (2,475; 95% CI: 1,651–3,783).

**Table 3:** Estimated number of people living with undiagnosed HIV^a^ with CD4 < 350 cells/mm^3^ or CD4 < 200 cells/mm^3^, by main characteristics and year, Italy, 2012–2014

	2012		2013		2014	
	Point estimate (n)	95% CI	Point estimate (n)	95% CI	Point estimate (n)	95% CI
**Undiagnosed with CD4 < 350 cells/mm^3^**						
**Total population**	**6,028**	**5,090***–***7,826**	**6,156**	**4,891***–***8,517**	**5,899**	**4,882***–***7,786**
**Sex**						
Women	1,230	1,021*–*1,690	1,200	650*–*2,051	1,017	499*–*1,790
Men	4,799	3,998*–*6,323	4,961	3,874–7,085	4,893	3,992*–*6,568
**Age group (years)**						
15–24	148	29*–*349	267	40*–*589	218	32*–*500
25–34	1,483	811*–*2,510	1,593	849*–*2,749	1,283	671*–*2,171
35–44	1,963	1,255*–*3,074	1,935	1,224*–*3,095	1,798	1,047*–*2,958
45–54	1,393	828*–*2,262	1,354	750*–*2,316	1,294	672*–*2,277
≥ 55	932	459*–*1,670	965	458*–*1,752	1,233	640*–*2,149
**HIV exposure group**						
PWID	413	132*–*868	304	104*–*603	213	55*–*457
Heterosexual women	1,039	539*–*1,787	952	480*–*1,689	827	374*–*1,514
Heterosexual men	1,863	1,177*–*3,322	2,018	1,242*–*3,263	2,017	1,183*–*3,301
MSM	1,860	1,164*–*2,937	2,050	1,255*–*3,279	2,115	1,292*–*3,395
Other/NA	835	376*–*1,554	1,037	273*–*2,268	868	306*–*1,701
**Nationality**						
Italian	4,386	3,382*–*6,101	4,703	3,617*–*6,617	4,178	3,128*–*5,922
Non-Italian	1,644	960*–*2,668	1,441	781*–*2,497	1,738	972*–*2,926
**Geographical area**						
North	3,063	2,203*–*4,462	2,924	2,025*–*4,414	2,475	1,651*–*3,783
Central	1,899	947*–*3,310	2,027	963*–*3,636	1,838	943*–*3,109
South	1,212	728*–*1,948	1,320	763*–*2,194	1,555	940*–*2,511
**Undiagnosed with CD4 < 200 cells/mm3**						
**Total population**	**2,467**	**2,052***–***3,145**	**2,456**	**2,027***–***3,151**	**2,524**	**2,075***–***3,246**
**Sex**						
Women	457	326*–*644	553	341*–*860	416	247*–*656
Men	2,012	1,650*–*2,590	1,903	1,544*–*2,472	2,114	1,714*–*2,755
**Age group (years)**						
15–24	96	27*–*203	81	18*–*178	65	8*–*155
25–34	461	272*–*731	442	251*–*709	358	196*–*587
35–44	810	546*–*1,215	826	547*–*1,247	812	530*–*1,236
45–54	638	423*–*952	654	421*–*1,002	667	421*–*1,033
≥ 55	406	238*–*645	419	251*–*664	569	345*–*896
**HIV exposure group**						
PWID	149	58*–*283	122	47*–*232	133	42*–*265
Heterosexual women	378	219*–*602	415	241*–*663	345	196*–*554
Heterosexual men	808	550*–*1,123	859	578*–*1,290	889	585*–*1,354
MSM	761	514*–*1,123	734	485*–*1,103	787	523*–*1,183
Other/NA	379	188*–*657	403	156*–*760	503	232*–*892
**Nationality**						
Italian	1,770	1,446*–*2,285	1,794	1,455*–*2,334	1,920	1,558*–*2,494
Non-Italian	701	452*–*1,070	674	417*–*1,047	590	350*–*942
**Geographical area**						
North	1,349	1,053*–*1,817	1,214	921*–*1,674	1,194	899*–*1,652
Central	531	284*–*877	729	417*–*1,171	768	421*–*1,254
South	517	341*–*777	520	335*–*795	568	369*–*866

CI: confidence interval; MSM: men who have sex with men; NA: not available; PWID: people who inject drugs.

^a^ Estimates adjusted for reporting delays and missing values.

The estimated number of people living with undiagnosed HIV infection and CD4 count < 200 cells/mm^3^ in Italy was 2,467 (95% CI: 2,052–3,145) in 2012, 2,456 (95% CI: 2,027–3,151) in 2013, and 2,524 (95% CI: 2,075–3,246) in 2014 (Table 3). Also for the undiagnosed people with CD4 count < 200 cells/mm^3^, the highest estimates were in men, those living in the North and in MSM.

Among the undiagnosed people living with HIV and with low CD4 count, the proportion of those with CD4 count < 200 cells/mm^3^ was 40.9% in 2012, 39.9% in 2013, and 42.8% in 2014. These proportions were similar according to all characteristics from 2012 to 2014.

### Characteristics of people undiagnosed and newly diagnosed with HIV and with low CD4 count in 2014

In Table 4, for the year 2014, main characteristics of people undiagnosed and newly diagnosed with HIV and with a low CD4 count are compared. The characteristics of those newly diagnosed and with a low CD4 count were similar to those of people with undiagnosed HIV and with a low CD4 count.

**Table 4:** Proportions of new HIV diagnoses, undiagnosed and yearly diagnosed fraction among people living with HIV and with low CD4 count^a^ by main characteristics, Italy, 2014

	New diagnoses with CD4 < 350 cells/mm^3^		Undiagnosed people with CD4 < 350cells/mm^3^		YDF in people with CD4 < 350 cells/mm^3^		New diagnoses with CD4 < 200 cells/ mm^3^		Undiagnosed people with CD4 < 200 cells/mm^3^		YDF in people with CD4 < 200 cells/mm^3^	
	n	%	n	%	%	95% CI	n	%	n	%	%	95% CI
**Total population**	1,529	100.0	5,899	100.0	20.6	16.4–23.8	998	100.0	2,524	100.0	28.3	23.5–32.5
**Sex**												
Women	344	22.5	1,017	17.2	25.3	16.1–40.8	216	21.6	416	16.4	34.2	24.8–46.7
Men	1,185	77.5	4,893	82.8	19.5	15.3–22.9	782	78.4	2,114	83.6	27.0	22.1–31.3
**Age group (years)**												
15–24	76	5.0	218	3.7	25.9	13.2–70.4	36	3.6	65	2.6	35.6	18.8–81.8
25–34	326	21.3	1,283	22.0	20.3	13.1–32.7	163	16.3	358	14.5	31.3	21.7–45.4
35–44	501	32.8	1,798	30.9	21.8	14.5–32.4	328	32.9	812	32.9	28.8	21.0–38.2
45–54	380	24.8	1,294	22.2	22.7	14.3–36.1	294	29.5	667	27.0	30.6	22.2–41.1
≥ 55	246	16.1	1,233	21.2	16.6	10.3–27.8	177	17.7	569	23.0	23.7	16.5–33.9
**HIV exposure group**												
PWID	65	4.3	213	3.5	23.4	12.5–54.2	48	4.8	133	5.0	26.5	15.3–53.3
Heterosexual women	308	20.1	827	13.7	27.1	16.9–45.2	195	19.5	345	13.0	36.1	26.0–49.9
Heterosexual men	486	31.8	2,017	33.4	19.4	12.8–29.1	347	34.8	889	33.5	28.1	20.4–37.2
MSM	539	35.2	2,115	35.0	20.3	13.7–29.4	306	30.7	787	29.6	28.0	20.6–36.9
Other/NA	131	8.6	868	14.4	13.1	7.2–30.0	102	10.2	503	18.9	16.9	10.3–30.5
**Nationality**												
Italian	1,105	72.6	4,178	70.6	20.9	15.7–26.1	738	74.3	1,920	76.5	27.8	22.8–32.1
Non-Italian	418	27.4	1,738	29.4	19.4	12.5–30.1	255	25.7	590	23.5	30.2	21.3–42.1
**Geographical area**												
North	895	58.5	2,425	42.2	26.6	19.1–35.2	570	57.1	1,194	47.1	32.3	25.7–38.8
Central	230	15.1	1,838	31.3	11.1^b^	6.9–19.6	156	15.6	768	30.4	16.9^b^	11.1–27.0
South	404	26.4	1,555	26.5	20.6	13.9–30.1	272	27.3	568	22.5	32.4	23.9–42.4

MSM: men who have sex with men; NA: not available; PWID; people who inject drugs; YDF: yearly diagnosed fraction.

^a^ Defined as CD4 < 350 cells/mm^3^ and CD4 < 200 cells/mm^3^.

^b^ Data in this table are underestimated, as in the Central area new diagnoses with missing CD4 count was at 60%.

The YDF is expressed as the yearly number of new diagnoses / (yearly number of new diagnoses + estimated number of undiagnosed people living with HIV).

Many people undiagnosed and with CD4 count < 350 cells/mm^3^ were men and older than 35 years, while a third were MSM and, another third were heterosexual men. About a third were born abroad and nearly half resided in the North of Italy. Similarly, men (MSM and heterosexual men), people older than 35 years, and those living in the North were among those most represented among undiagnosed people with HIV and with CD4 count < 200 cells/mm^3^ (Table 4).

In Table 4 also shows the YDFs by main characteristics and CD4 count. The YDF was 20.6% (95% CI: 16.4–23.8%) among people with CD4 count < 350 cells/mm^3^; the highest proportion was observed among heterosexual women (27.1%; 95% CI: 16.9–45.2%) and among people living in the North (26.6%; 95% CI: 19.1–35.2%).The YDF among people with CD4 count < 200 cells/mm^3^ was 28.3% (95% CI: 23.5–32.5%); once again, the highest proportion was observed among heterosexual women (36.1%; 95% CI: 26.0–49.9%). Similar results for the previous years (2012 and 2013) were observed (data not shown).

### Prevalence of people living with undiagnosed HIV and with low CD4 cell count in 2014

**Figure 1:**
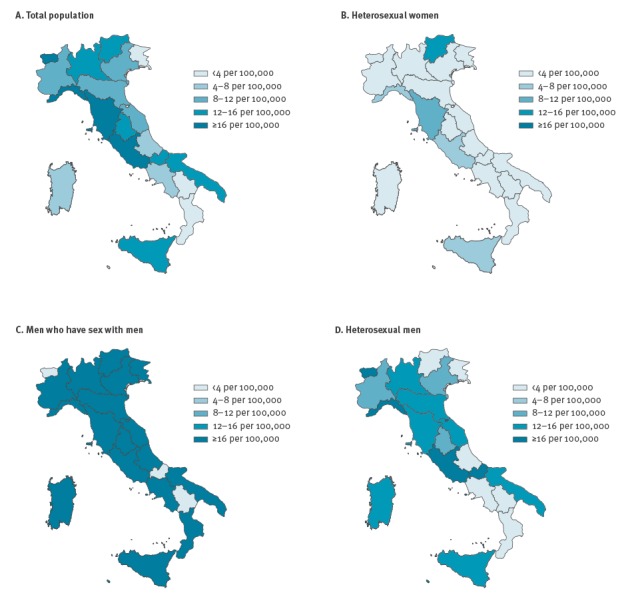
Prevalence rates of people living with undiagnosed HIV and with low CD4 cell count^a^ by HIV exposure group^b^ and region^c^, Italy, 2014

Figure 1 shows the prevalence of people with undiagnosed HIV and with CD4 < 350 cells/mm^3^, calculated as a rate per 100,000 adult residents. Overall, this rate was 11.3 (95% CI: 9.3–14.9) per 100,000 residents older than 15 years. The prevalence of people with undiagnosed HIV varied between the different Italian regions from 0.7 per 100,000 (Calabria) to 20.8 per 100,000 adults (Liguria); North and Central areas showed higher rates of those undiagnosed with a low CD4 count (Figure 1A ).

MSM: men who have sex with men.

^a^ CD4 count < 350 cells/mm^3^

^b^ The undiagnosed rates were calculated as follows: total population (panel A) – number of all undiagnosed with CD4 < 350 cells/mm^3^ divided by the number of residents in each region, multiplied by 100,000; Italian average: 11.3 (95%CI:9.3-14.9) per 100,000 residents. Heterosexual women (panel B) – number of undiagnosed heterosexual women with CD4 < 350 cells/mm^3^ divided by the number of women resident in each region, multiplied by 100,000; Italian average: 3.0 (95%CI: 1.4-5.6) per 100,000 heterosexual women residents. MSM (panel C) – number of undiagnosed MSM with CD4 counts < 350 cells/mm^3^ divided by the number of MSM (3% of men) resident in each region, multiplied by 100,000; Italian average: 280.4 (95%CI: 173.3–450.2) per 100,000 MSM residents. Heterosexual men (panel D) – number of undiagnosed heterosexual men with CD4 counts < 350 cells/mm^3^ divided by the number of heterosexual men (97% of men), resident in each region, multiplied by 100,000; Italian average: 8.3 (95%CI:4.9-13.6) per 100,000 heterosexual men residents.

^c^ For regions (one in the Central area and one in the North area) that did not collect data on the clinical stage and CD4 count, the number of undiagnosed was estimated assuming the distribution of clinical stage and CD4 count observed at national level (Table 2).

Figure 1 (Panel B–D) shows the regional prevalence rates of undiagnosed people with HIV and with low CD4 cell count by HIV exposure groups. For heterosexual women the prevalence rate was 3.0 (95% CI: 1.4–5.6) per 100,000 women; the regional rates ranged from 0.1 (Friuli) to 15.6 (Trentino Alto Adige). Most of the Italian regions (15 regions) had prevalence rates ranging from 2.0 to 4.0 per 100,000 women (Figure 1B). For MSM the rate at national level was 280.4 (95% CI: 173.3 – 450.2) per 100,000 MSM, for heterosexual men it was 8.3 (95% CI: 4.9–13.6) per 100,000 heterosexual men. The prevalence rates among MSM ranged from 6.2 (Basilicata, Molise, and Valle d’Aosta) to 450.6 (Liguria); almost all regions showed rates higher than 16 per 100, 000 (Figure 1C), in particular five regions showed a regional rate higher than 300.0 per 100,000 MSM (Umbria, Sicilia, Toscana, Lombardia, and Liguria) (results not shown in the figure). The regional rates varied for heterosexual men from 0.4 (Basilicata, and Friuli) to 16.1 (Valle d’Aosta) per 100,000 heterosexual men; almost half of the Italian regions (nine regions) had an estimated prevalence rate higher than 8.0 per 100,000 heterosexual men (Figure 1D).

The annual rate of new diagnoses in Italy was 6.1 per 100,000 adult residents in 2014, ranging from 2.0 (Calabria region) to 11.1 (Lazio region) [5] (data not shown).

**Figure 2:**
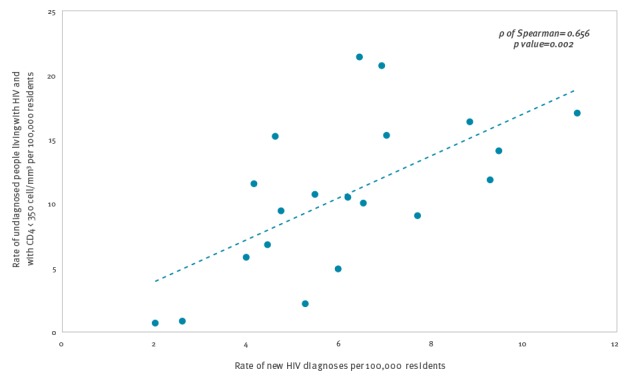
Correlation between the prevalence rates of undiagnosed people living with HIV and with low CD4 count^a^ and new HIV diagnoses rates, Italy, 2014

^a^ CD4 count < 350 cells/mm^3^

Figure 2 shows the relationship between the prevalence rate of undiagnosed HIV infection with CD4 count < 350 cells/mm^3^, and the rate of new HIV diagnoses in the 20 Italian regions. A positive correlation (ρ Spearman = 0.66; p value = 0.002) showed that regions with higher rate of new diagnoses also were the regions with a higher rate of undiagnosed people.

## Discussion

Estimating the number of people living with undiagnosed HIV and with a low CD4 count enables the identification of determinants for a delayed access to care. We estimated the number of people with HIV and with a low CD4 count in Italy who are not yet diagnosed using an easy, reproducible, and validated model [13]. The strength of this study is that it provided estimates of demographic characteristics of undiagnosed people with HIV. The average yearly number of people living with undiagnosed HIV infection and CD4 < 350 cells/mm^3^ was 6,000 over the period 2012 to 2014, with a similar pattern across the years. The estimate of people with low CD4 count corresponded to 40% of the total number of people (including asymptomatic) with undiagnosed HIV infection in Italy (i.e. 15,000) [10]. The same proportion of people (40%) with CD4 count < 350 cells/mm^3^ was found in France for the estimated undiagnosed people with HIV in 2010 [22]. Our numbers indicate there are a substantial number of people with undiagnosed HIV in Italy who need to be treated immediately. Failure to diagnose these individuals will result in greater morbidity and mortality for them, risk of onward transmission and greater costs accrued for the health system.

Focusing on the most recent year analysed, the prevalence of undiagnosed HIV infections was 11.3 per 100,000 adults in the resident population in 2014, ranging from 0.7 to 20.8 for different regions. Differences in regional prevalence could be attributed to factors, such as (i) different spread of HIV infection [5,15], (ii) different levels of HIV risk awareness [23,24], and (iii) the risk groups prevalent in each region.

This is in line with a cross-sectional study [20] that indicated a higher prevalence of people diagnosed and linked to care in northern Italy. Moreover, despite IDCs being well distributed throughout Italy, surveillance data indicates higher numbers of new diagnoses of HIV infection and AIDS, as well as of HIV-positive people under treatment, in the North [5,15].

Different levels of HIV risk awareness were confirmed in a respective study, which showed that people living in the North were less aware of HIV risk factors compared with those in the Centre and South of Italy [23]. Furthermore, a study showed that regional differences of HIV risk awareness seem to be correlated with different socioeconomic factors and lifestyles existent in North and South Italy (unpublished data).

Differences in regional prevalences of undiagnosed HIV infection were very similar to those observed among HIV-positive people diagnosed that were linked to care [15] as well as to differences observed among new HIV diagnoses across the Italian regions [5]. These findings confirm that, at least in Italy, regions with high rates of new diagnoses also encompass a high proportion of both diagnosed and undiagnosed people [5,15]. This highlights the importance of the regional differences in the spread of HIV infection that can be observed at a wider level across European countries as well as within the United States [25,26].

In addition, the highest prevalence of undiagnosed HIV infection was observed among MSM in whom it was 280 per 100,000 MSM, whereas among heterosexual men it was 8 per 100,000 heterosexual men, and among heterosexual women it was 3 per 100,000 female residents, with large differences across the Italian regions. Even though MSM have been reported to have high HIV testing rates compared with other key populations in high-income countries [24,27-29], as well as, the highest perception of the risk of HIV infection [23,24], the study findings show that they account for the highest number (2,115) and the highest proportion (35%) of undiagnosed people with a low CD4 count in 2014. MSM in Italy are also the subgroup most represented (nearly 50%) among the total population of undiagnosed HIV people (including asymptomatic), as estimated by Mammone et al. [10]. This could be attributed to a high rate of new infections in this group during the most recent years [1,30-32] combined with a large number of undiagnosed people who contribute to ongoing transmission [10,22,33]. In addition, a high HIV prevalence and a high proportion of MSM with undiagnosed HIV could be attributed to high levels of sexual activity and to some risk behaviours for sexual transmission of HIV [34]. Therefore, test-seeking behaviour should be encouraged and voluntary counselling and testing made more accessible in Italy, a country where the stigma against HIV and homosexuality may still be prevalent [20,35].

Focusing on the most recent year in our analysis, a high proportion of undiagnosed people with low CD4 count was reported among heterosexual men (33.4%), whereas in other Italian studies this population accounted for a quarter of the total undiagnosed (including those asymptomatic [10]), and a quarter of new diagnoses reported to the INHS [5]. The higher proportion of heterosexual men among undiagnosed with a low CD4 count could depend, partly, on the fact that heterosexuals were more likely to have a longer undiagnosed interval (time lag from infection to HIV diagnosis) as shown in other studies worldwide [8,36-38]. In Italy, Mammone et al. [37] estimated that heterosexuals had a far longer lag from infection to HIV diagnosis compared with MSM (7.7 vs 3.7 years).

We found a YDF of 20.6% which was similar to that reported recently by Sasse [18] on the total HIV population in the European countries. Among heterosexual women the YDF was the highest (27.1%) compared to the other groups, suggesting a more frequent access to HIV testing, likely facilitated from routine screening during pregnancy in this population [5,39]. This result may mean a certain degree of success with regard to testing in this group. The highest YDF (26.6%) among people living in the North compared to the other areas may be an indicator of the wider availability of IDCs and HIV testing services in this area [40]. A higher detection could represent a more efficient and therefore better surveillance system.

This study has some limitations. First, we assumed that people with HIV who develop AIDS, or other HIV-related symptoms, will almost certainly present for care, and as consequence, will be all diagnosed with HIV and notified to the surveillance system (assumption of London method) [12,13]. However, the assumption of the London method can be considered acceptable for our study, as HIV testing and access to care are free in all IDCs and the proportion of people living with HIV who do not attend the IDCs should be reasonably low. Another limitation was the assumption that CD4 counts in those where the information was not available was the same as in those with available information. This assumption was supported by other studies conducted on the Italian HIV Surveillance data [10]. The missing CD4 count information, In the Italian national HIV surveillance data, mainly in the Central regions, may make the estimates less robust. However, in the remaining areas the proportion of missing data were lower than 10%.

In terms of the reporting delay we assumed a constant decrease over the 3 years. This had a small impact on the estimates as it was sufficiently low. Other limitations which may have a considerable impact on the eventual estimates include the effect of new testing strategies, the changes over time in the reporting of data, and the different quality of data in the surveillance systems of all the regions.

## Conclusions

About 6,000 HIV-positive people with low CD4 counts, remained annually undiagnosed between 2012 and 2014 in Italy. This indicates that ca 40% of the 15,000 total undiagnosed people living with HIV in Italy were in immediate need of diagnosis, linkage to care and antiretroviral treatment in order to avert high HIV-related morbidity, mortality and healthcare costs.

The majority of those with undiagnosed HIV and with low CD4 counts were MSM and heterosexual men, and there were large differences in prevalence of undiagnosed HIV infections with low CD4 across the Italian regions. These findings highlight the importance of improving HIV testing availability, with a focus on men, in order to diagnose and provide treatment to those living with undiagnosed HIV in Italy.

## Regional representatives of the National HIV Surveillance System

National HIV Surveillance System include: Abruzzo: Manuela Di Giacomo, Viviana Faggioni, Luigi Scancella; Basilicata: Francesco Locuratolo, Gabriella Cauzillo; Calabria: Anna Domenica Mignuoli, Daniele Giuseppe Chirico; Campania: Guglielmo Borgia; Emilia Romagna: Erika Massimiliani; Friuli Venezia Giulia: Tolinda Gallo, Cinzia Braida; Lazio: Vincenzo Puro, Paola Scognamiglio, Alessia Mammone; Liguria: Giancarlo Icardi, Piero Luigi Lai; Lombardia: Maria Gramegna, Liliana Coppola, Alessandra Piatti, Annamaria Rosa, Danilo Cereda; Marche: Fabio Filippetti; Molise: Alessandra Prozzo; Piemonte: Chiara Pasqualini; Provincia Autonoma di Bolzano: Peter Mian, Oswald Moling, Leonardo Pagani; Provincia Autonoma di Trento: Paolo Lanzafame, Lucia Collini, Danila Bassetti; Puglia: Maria Chironna; Sardegna: Maria Antonietta Palmas; Sicilia: Gabriella Dardanoni; Toscana: Fabio Voller, Monia Puglia, Lucia Pecori; Umbria: Anna Tosti, Rita Papili; Valle d’Aosta: Mauro Ruffier, Marina Giulia Verardo, Elisa Francesca Echarlod, Saveria Amoroso; Veneto: Francesca Russo, Filippo da Re.
